# Differentially Expressed Functional LncRNAs in Human Subjects With Metabolic Syndrome Reflect a Competing Endogenous RNA Network in Circulating Extracellular Vesicles

**DOI:** 10.3389/fmolb.2021.667056

**Published:** 2021-08-17

**Authors:** Yongxin Li, Yu Meng, Yuanhang Liu, Andre J. van Wijnen, Alfonso Eirin, Lilach O. Lerman

**Affiliations:** ^1^ Department of Vascular Surgery, The Affiliated Hospital of Qingdao University, Qingdao, China; ^2^ Division of Nephrology and Hypertension, Mayo Clinic, Rochester, MN, United States; ^3^ Central Laboratory, The Fifth Affiliated Hospital of Jinan University, Heyuan, China; ^4^ Department of Nephrology, The First Affiliated Hospital of Jinan University, Guangzhou, China; ^5^ Health Sciences Research and Division of Biomedical Statistics and Informatics, Mayo Clinic, Rochester, MN, United States; ^6^ Department of Orthopedic Surgery, Mayo Clinic, Rochester, MN, United States

**Keywords:** metabolic syndrome, extracellular vesicles, lncRNAs, competing endogenous RNA, circulation

## Abstract

Metabolic syndrome (MetS), a collective cluster of disease risk factors that include dyslipidemia, obesity, inflammation, hypertension, and insulin resistance, affects numerous people worldwide. Accumulating studies have shown that long non-coding RNAs (lncRNAs) serve as competing endogenous RNAs (ceRNAs) to play essential roles in regulating gene expression in various diseases. To explore the role of lncRNAs as ceRNAs in MetS, we examined a MetS-associated network in circulating extracellular vesicles (EVs) collected from the systemic blood of MetS and control patients (*n* = 5 each). In total, 191 differentially expressed lncRNAs, 1,389 mRNAs, and 138 miRNAs were selected for further analysis. Biological processes and pathway functional enrichment analysis were performed based on the Database for Annotation, Visualization, and Integrated Discovery (DAVID). The lncRNA/mRNA/miRNA ceRNA network was constructed by Cytoscape v3.8 based on the DE-RNAs and included 13 lncRNAs, 8 miRNAs, and 64 mRNAs. MetS patients showed elevated body weight, glucose, blood pressure, insulin, liver injury, and inflammatory marker levels. We found that lncRNAs reflect a ceRNA network that may regulate central cellular processes and complications of MetS, including cancer. These findings suggest that MetS alters the interactions among the ceRNA network components in circulating EVs and that this cargo of circulating EVs may have potential translational ramifications for MetS.

## Introduction

Metabolic syndrome (MetS) is a constellation of metabolic abnormalities associated with dyslipidemia, obesity, inflammation, insulin resistance, and hypertension that leads to metabolic diseases such as obesity, type 2 diabetes, and hepatic and cardiovascular diseases, affecting numerous people worldwide (Sung et al., 2018). The molecular mechanisms involved in this syndrome have not been fully elucidated but likely include aberrant activation of multiple signaling pathways.

Extracellular vesicles (EVs), cell-derived lipid bilayer-enclosed vesicles of sub-micrometer sizes, are involved in cell–cell communication in a wide range of physiological and pathological processes *in vivo*. EVs are categorized into three main subgroups: exosomes, microvesicles, and apoptotic bodies, and are released by many types of cells (Crescitelli et al., 2013). Growing evidence indicates that EVs shuttle cell-derived biological molecules such as nucleic acids (DNA, mRNA, lncRNA, and miRNA), proteins, and lipids, reflecting the status of their parental cells and serving in inter-cellular communication. Studies have found that EVs can modulate cellular pathways and tissue metabolism by altering transcription profiles in recipient cells (Crewe et al., 2018).

Recently, studies have implicated non-coding RNAs (ncRNAs), which have no significant protein‐coding potential, in the development of MetS (Lan et al., 2016; Liu et al., 2016). These ncRNAs include short non-coding micro-RNAs (miRNAs), long non-coding RNAs (lncRNAs), and other classic ncRNAs. In 2011, Salmena et al. presented a novel regulatory hypothesis whereby RNA transcripts can communicate with and regulate each other via miRNA response elements (MREs), thereby serving as competing endogenous RNAs (*ceRNAs*) (Salmena et al., 2011). Based on this hypothesis, lncRNAs, non-coding transcripts >200 nucleotides long, can act as sponges by competing for specific miRNAs, thus de-repressing their target genes. Both lncRNAs and miRNAs have been demonstrated to play pivotal roles in regulating the physiological and pathophysiological processes of MetS (Lan et al., 2016; Assmann et al., 2020).

Our group recently showed that EVs derived from pig adipose tissue–derived mesenchymal stem cells are selectively packed with miRNAs, mRNAs, and proteins, which have the capacity to alter selective pathways in recipient cells, and that MetS alters the cargo of genes, proteins, and miRNAs packed in these EVs (Eirin et al., 2016; Meng et al., 2018). EVs are also shed by different cell types into the systemic circulation, and their cargo might reveal the status of their parent cells and modes of regulation of remote target cells. Yet, the content of lncRNAs in circulating human EVs or about their potential interactions with other cargo components have not been well-defined. Furthermore, the network of MetS-specific ceRNAs and underlying interactions remains unclear. Identifying lncRNA, miRNA, and mRNA cargo of circulating EVs, and elucidating alterations due to MetS, could illuminate their potential as biomarkers of tissue and organ injury.

Therefore, this study was designed to identify the differential expression of lncRNAs, miRNAs, and mRNAs and in turn construct the lncRNA-associated ceRNA network in circulating EVs obtained from human subjects with MetS. We tested the hypothesis that MetS alters the interaction among the ceRNA network components.

## Materials and Methods

### Patient Population

MetS patients and healthy subjects (*n* = 5 for each group) were recruited from the First Hospital Affiliated to Jinan University (Guangdong, China). Consistent with the Declaration of Helsinki, our study was approved by the Institutional Research Ethics Committee, and written informed consent was obtained from all subjects. All participants were reviewed for medical history.

Based on criteria of the International Diabetes Federation (NCEP (2001)), inclusion criteria for MetS patients included diagnosis of MetS and age>18 years. The diagnosis of MetS was centered on obesity (body mass index [BMI] >30 kg/m^2^) and two or more of the following: abnormal lipid metabolism (levels of HDL cholesterol <40 mg/dl in males and <50 mg/dl in females, triglycerides ≥150 mg/dl), diastolic blood pressure ≥85 mmHg or systolic blood pressure ≥130 mmHg; fasting glucose concentration ≥100 mg/dl or previously diagnosed hypertension or type 2 diabetes. Exclusion criteria included severe cardiac diseases, heavy smoking, drug abuse, cancer, or any severe systemic diseases.

Healthy controls were overall healthy individuals >18 years of age. Exclusion criteria for healthy controls included drug abuse, heavy smoking, or any significant disease.

Under fasting condition, blood samples were collected for assessment of metabolic, renal, and liver functions following routine procedures in the clinical laboratories of the First Hospital Affiliated to Jinan University. The estimated glomerular filtration rate (eGFR) was calculated by the Modification of Diet in Renal Disease Equation (Levey et al., 2009).

### Blood EV Harvesting

Using the exoRNeasy Serum/Plasma (Qiagen cat# 77044) assay, circulating EVs were isolated following the vendor’s instructions, and RNA was subsequently isolated with the miRNeasy serum/plasma advanced kit (Qiagen cat# 217204). Initially, thrombin was added to the plasma for 5 min, and the sample was centrifuged at 2,500×g for 15 min. After being mixed with precipitation buffer and incubated at 4°C for 60 min, the supernatant was centrifuged at 13,000×g for 5 min. The pellet was resuspended and lysed, and proteins were precipitated and removed. Isopropanol was added to the supernatant, which was then loaded onto the column. RNA was eluted following three washes and stored at −80°C.

### EV Characterization

Following the standards described by minimal information for studies of extracellular vesicles 2018 (MISEV2018) guidelines (Thery et al., 2018), EVs were characterized based on the expression of common EV (CD9, CD63, and CD81) protein markers (western blotting), transmission electron microscopy (TEM negative staining, JEOL 1200 EXII), and nanoparticle tracking analysis (NTA, NanoSight NS300) to assess the EV concentration and size distribution, as we have shown before (Meng et al. 2018).

### mRNA and lncRNA Sequencing Analysis

RNA sequencing was performed and analyzed as before (Meng et al., 2018). RNA libraries were prepared (TruSeq RNA Sample Prep Kit v2, Illumina, San Diego, United States) and loaded onto flow cells (8–10 pM) to generate cluster densities of 700,000/mm^2^. Using TruSeq SBS Kit version 3 and HCS v2.0.12 data collection software, the cells were sequenced on an Illumina HiSeq 2000 system. The generated data were analyzed using the MAPRSeq v.1.2.1 system and the Bioinformatics Core standard tool.

### MiRNA Sequencing and Data Analysis

Using the QIAseq Stranded Total RNA Kit, EV total RNA libraries were prepared and sequenced with an Illumina NGS system (MiSeq Personal Sequencer, NextSequence500, HiSeq 1000, HiSeq 1500, HiSeq 2000, HiSeq 2500, and GaIIx). Then, the data were analyzed with CLC (Biomedical) Genomics Workbench. Starting with unaligned FASTQ files, the workflow generates aligned BAMs and then both raw and normalized known mature miRNA expression counts. EdgeR2.6.2 was used for the differential expression analysis to identify miRNAs enriched or depleted in MetS-EVs compared to Lean-EVs.

### Validation of RNA-Seq Data

Several differentially expressed mRNAs (GYS1 and TRRAP), miRNAs (miR-4293), and lncRNAs (AC138720, AC093484, and AC007402) were selected for validation, and their expression in Lean- and MetS-EVs was measured by quantitative polymerase chain reaction (qPCR, PROMEGA.prcl).

### Construction of ceRNA Network

miRNA–mRNA interactions were downloaded from miRTarBase (v8.0) that contains experimentally validated miRNA targets (Huang et al., 2020). miRNA–lncRNA interactions were constructed based on DIANA-LncBase (Karagkouni et al., 2020) (v3) and miRcode (Jeggari et al., 2012) (v11). To focus on MetS-EVs compared to Lean-EVs, only DE-miRNAs, mRNAs, and lncRNAs were included in the ceRNA network. The potential ceRNA network was constructed based on the following criteria: 1) mRNA and lncRNA share the same miRNA. 2) There is significant positive correlation between mRNA and lncRNA (*p* < 0.05 and *r* > 0.3). 3)There is significant negative correlation between miRNA and target mRNA (*p* < 0.05 and *r* < −0.3). 4) There is significant negative correlation between miRNA and target lncRNA (*p* < 0.05 and *r* < −0.3). Accordingly, the ceRNA network was generated using Cytoscape (Shannon et al., 2003) (v3.8.0). Subsequent functional annotation-clustering analysis utilized the PANTHER database (http://www.pantherdb.org/) and DAVID 6.7.

### Statistical Analysis

Statistical analysis was performed using JMP 14.0 (SAS Institute, Cary, NC). Data are expressed as mean ± standard deviation. Comparisons between groups were performed using unpaired Student’s *t*-test and ANOVA. Non-parametric tests (Wilcoxon and Kruskal–Wallis) were used for data not following a Gaussian distribution. RNAs showing fold-change >1.4 in the MetS vs. the Lean group were considered upregulated (enriched), whereas those with fold-change <0.7 were considered downregulated (depleted). Statistical significance was accepted if *p* ≤ 0.05.

## Results

### Characterization of Lean and MetS Participants

Participants’ demographic, clinical, and laboratory characteristics are summarized in Table 1.

**TABLE 1:** Clinical, laboratory, and demographic data of Lean and MetS patients (*n* = 5 each).

Parameter	Lean	MetS
Age (years)	24.8 (21–29)	27.8 (24–32)
Sex (female/male)	2/3	2/3
Body mass index (kg/mm^2^)	19.4 ± 1.0	59.5 ± 13.7*
Systolic blood pressure (mmHg)	110.8 ± 11.9	152.6 ± 9.7*
Diastolic blood pressure (mmHg)	64.0 ± 5.9	96.2 ± 4.5*
Hemoglobin A1C (%)	5.3 ± 0.3	6.9 ± 1.0*
Total cholesterol (mmol/L)	4.4 ± 0.4	5.1 ± 0.4
Low-density lipoprotein (mmol/L)	1.7 ± 0.3	3.1 ± 0.5*
Blood urea nitrogen (mmol/L)	3.9 ± 0.6	5.6 ± 1.4*
eGFR (ml/min/1.73m^2^)	117.5 ± 39.8	192.6 ± 6.6*
Fasting blood sugar (mmol/L)	4.8 ± 0.5	7.6 ± 2.0*
Insulin (mIU/L)	13.8 ± 3.8	38.6 ± 16.4*
C-peptide (ng/ml)	2.6 ± 0.6	5.7 ± 0.9*
Alanine aminotransferase (U/L)	31 ± 7.5	92 ± 27.7*
Aspartate aminotransferase (U/L)	26.8 ± 7.0	77 ± 33.1*
White blood cells 10^9/L*	5.05 ± 2.2	8.91 ± 2.5*
Plasma renin activity (ng/ml/h)	0.5 ± 0.1	4.5 ± 2.1*

eGFR: estimated glomerular filtration rate. *P<0.05 vs. Lean.

The two groups were similar with regard to age and sex, but BMI and blood pressure were markedly elevated in the MetS compared to the Lean group. Low-density lipoprotein, C-peptide, fasting blood sugar, insulin, and hemoglobin A1C levels were also elevated in MetS compared to Lean participants, consistent with the development of MetS. Additional abnormal laboratory findings included higher blood urea nitrogen (BUN) and eGFR in MetS suggesting renal hyperfiltration, higher white blood cell count suggesting inflammation, and elevated alanine aminotransferase (ALT) and aspartate aminotransferase (AST) indicating early liver injury in MetS.

### EV Characterization

**FIGURE 1:**
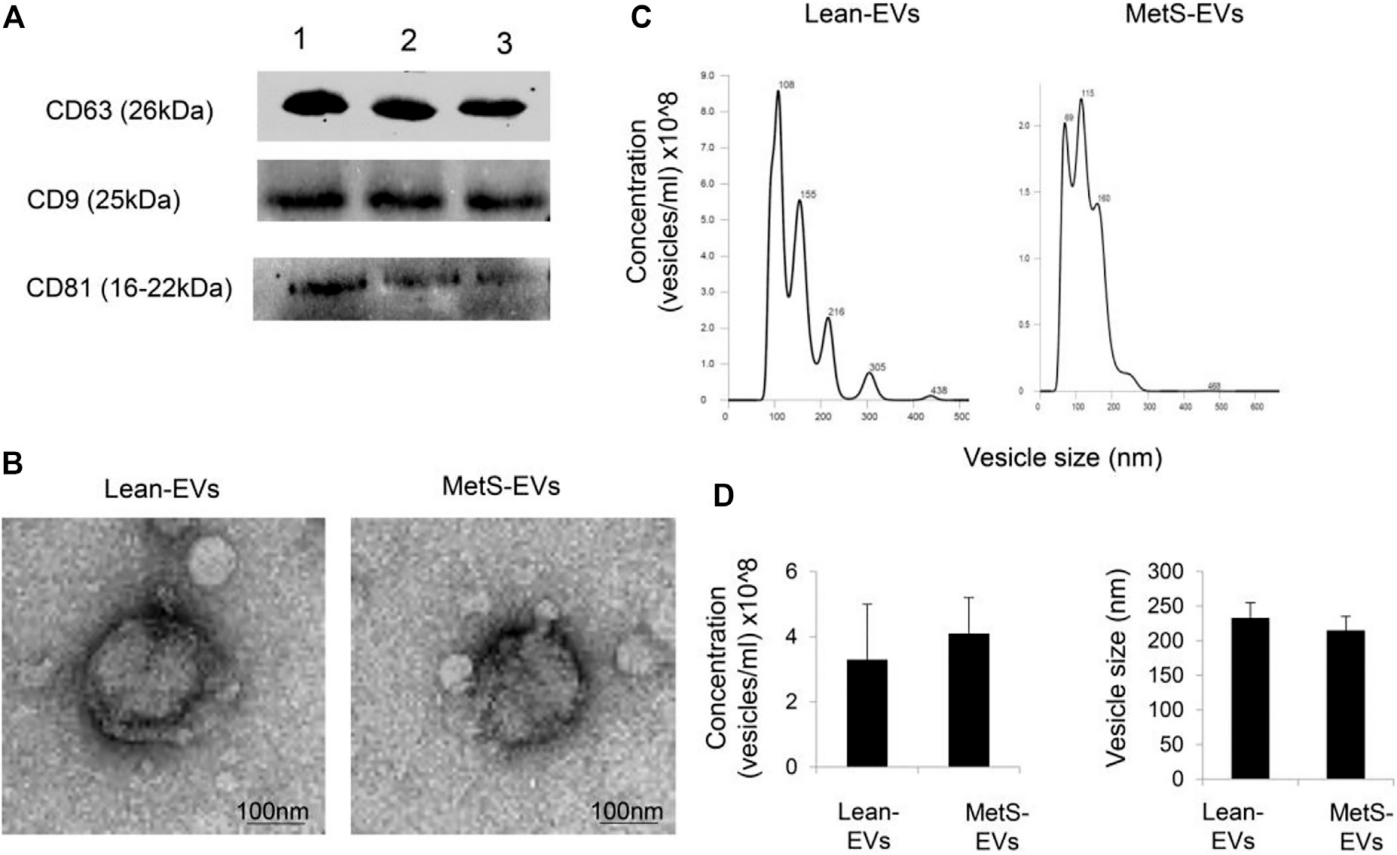
**(A)** Circulating EVs expressed common EV markers, including CD9, CD63, and CD81, by western blotting. **(B)** Transmission electron microscopy showing circulating Lean- and MetS-EVs exhibiting the classic “cup-like” morphology. **(C)** Representative size-distribution curve of Lean- and MetS-EVs by nanoparticle tracking analysis (NTA). **(D)** Quantification showed comparable EV concentration and size by NTA between the groups (Student’s *t*-test).

Circulating EVs expressed common EV markers, including CD9, CD63, and CD81 (Figure 1A), and exhibited the classic “cup-like” morphology on transmission electron microscopy (Figure 1B). The concentration and size of circulating EVs were similar between the groups (Figures 1C,D).

### Identifying DE-RNAs in MetS and Control Samples

**FIGURE 2:**
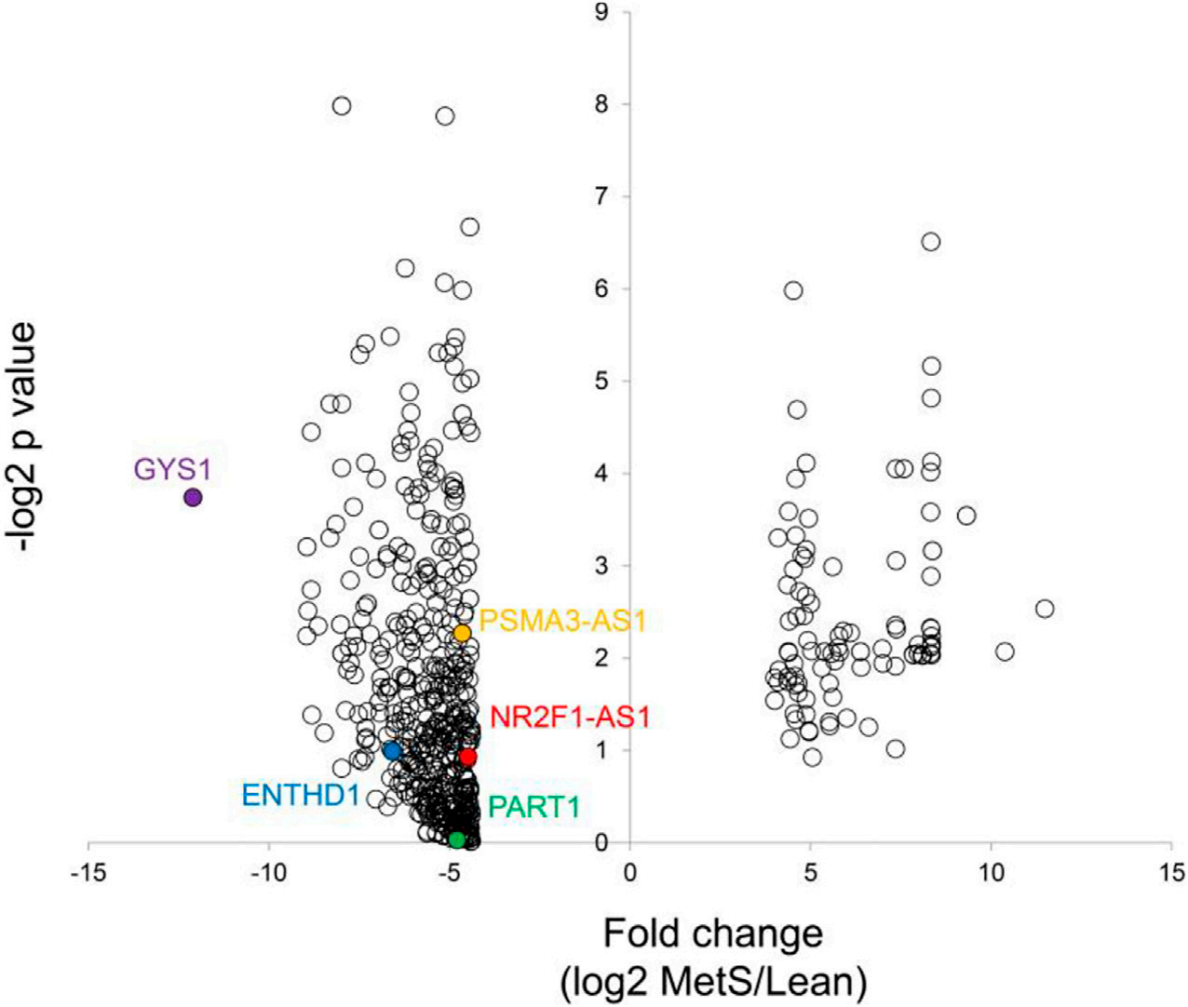
Volcano plot of differentially expressed mRNAs in circulating MetS-EVs versus Lean-EVs.

**FIGURE 3:**
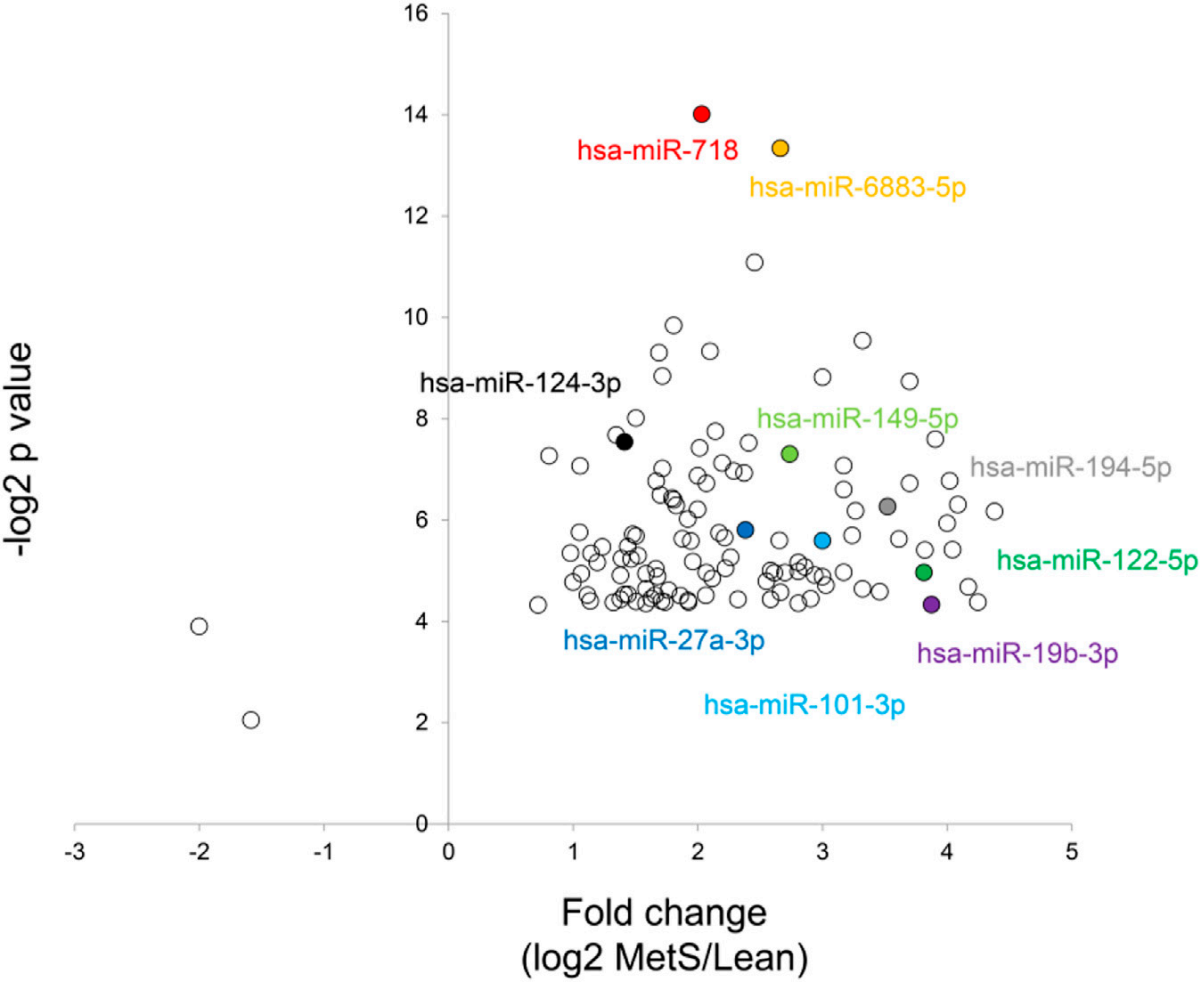
Volcano plot of differentially expressed miRNAs in circulating MetS-EVs versus Lean-EVs.

**FIGURE 4:**
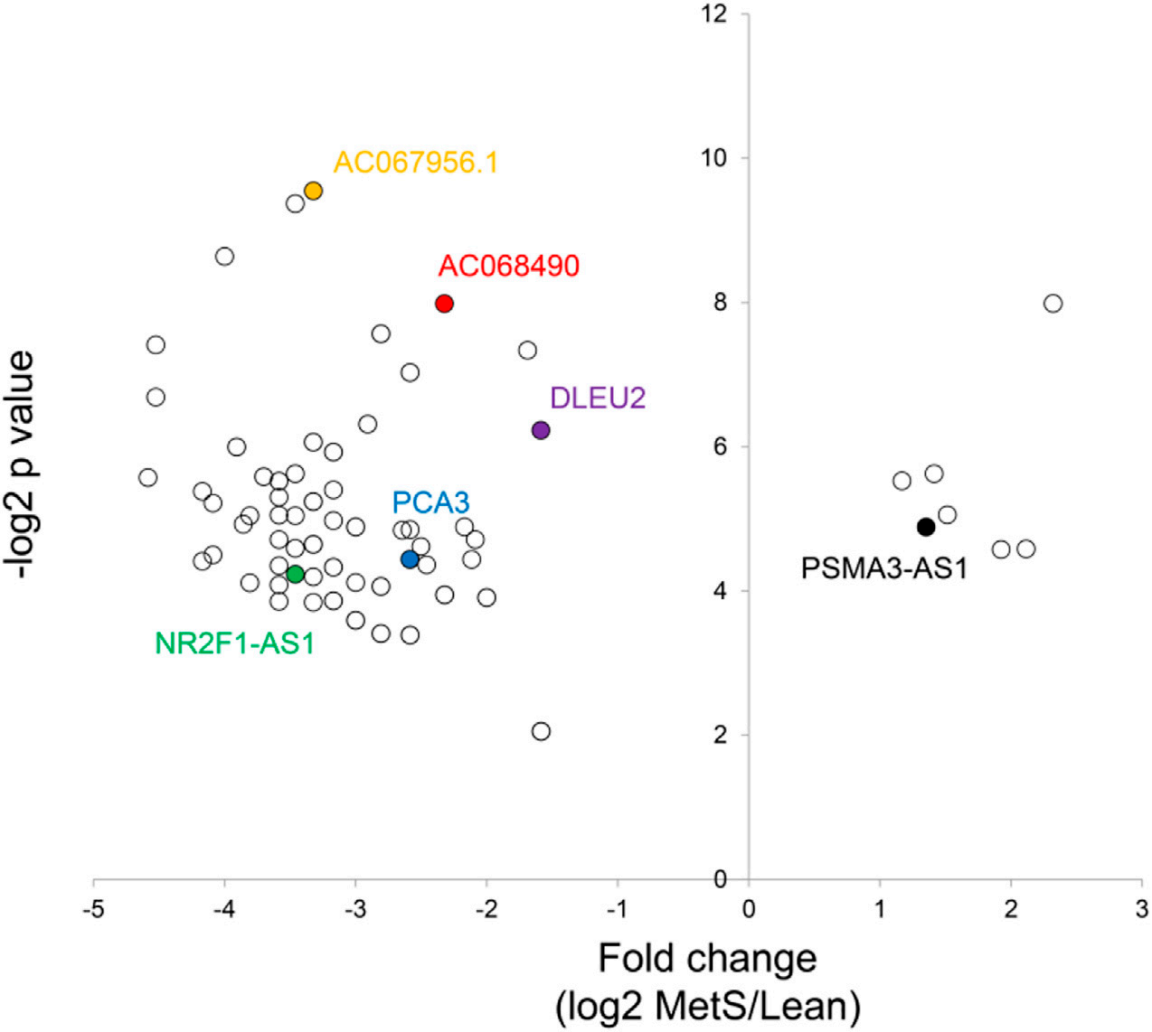
Volcano plot of differentially expressed lncRNAs in circulating MetS-EVs versus Lean-EVs.

The annotated genes amounted to >32,000. Of these, 106 (0.3%) were upregulated, while 1,283 (4.0%) mRNAs were downregulated in circulating MetS-EVs compared to Lean-EVs (Figure 2). Among the top 30 DE-mRNAs are ENTHD1 and *GYS1*, which are involved in endocytosis and glucose metabolism, respectively. Moreover, 1,515 miRNAs were annotated, of which 136 (8.9%) distinct miRNAs were selectively enriched and 2 (0.1%) were downregulated in MetS-EVs (Figure 3). Contrarily, 7 (0.1%) distinct lncRNAs were selectively enriched and 184 (2.3%) were downregulated in MetS-EVs compared to Lean-EVs, out of 8,051 annotated lncRNAs (Figure 4). Top DE-miRNAs included miR-718 that represses pro-inflammatory cytokines (Kalantari et al., 2017) and miR-688-5p that is involved in cell-cycle arrest and apoptosis (Lulla et al., 2017), whereas top DE-lncRNAs included AC068490 and AC067956, the function of which is yet unclear.

### Validation of RNA-Seq Data

**FIGURE 5:**
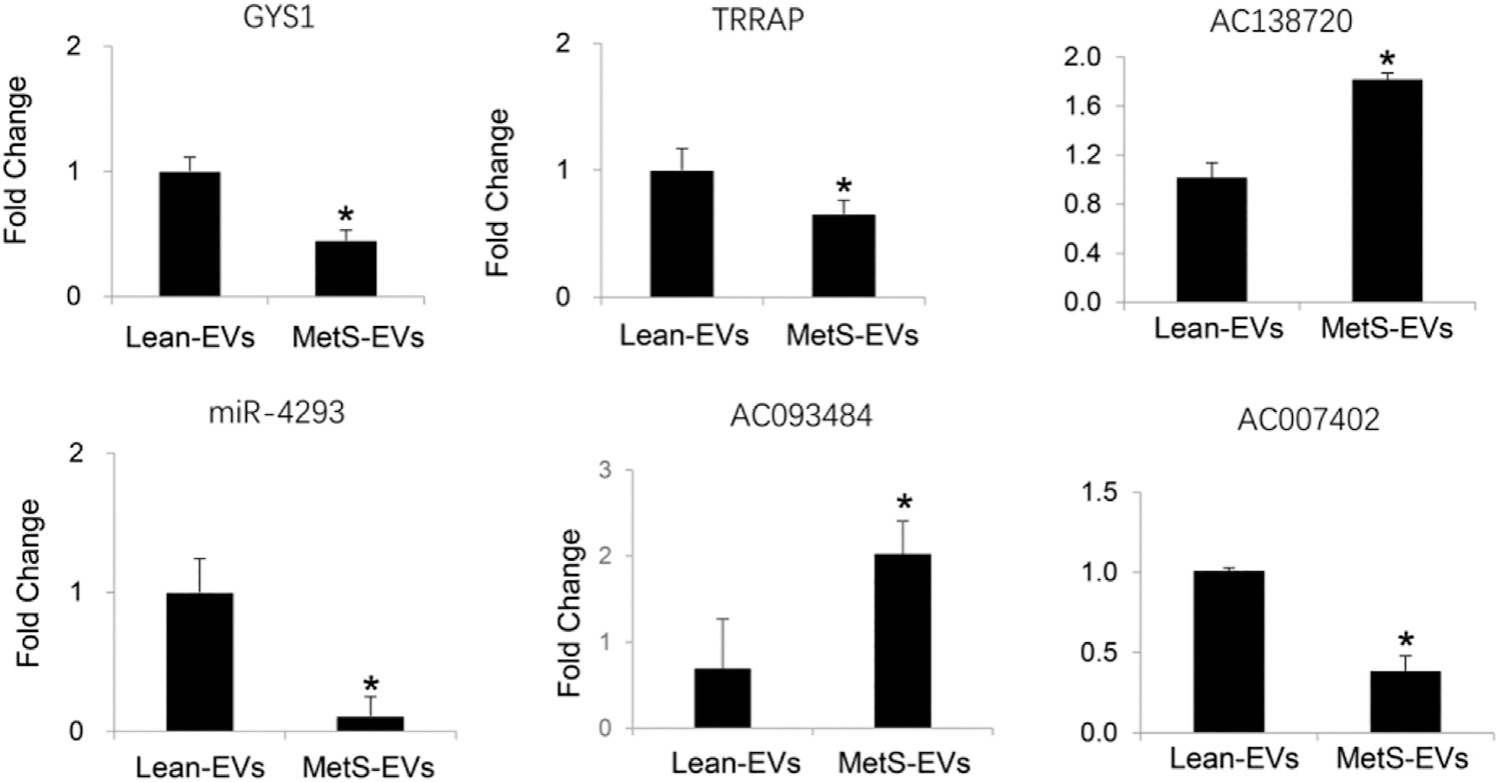
qPCR showing the expression of candidate mRNAs, miRNAs, and lncRNAs following the same patterns as the RNA-Seq findings (Student’s *t*-test). *p < 0.05 vs. Lean-EVs.

The expression of the candidate mRNAs (GYS1 and TRRAP), miRNAs (miR-4293), and lncRNAs (AC138720, AC093484, and AC007402) followed the same patterns as the proteomics findings (Figure 5).

### ceRNA Network Construction in MetS

To elucidate the roles of DE-lncRNA, DE-miRNA, and DE-mRNA in circulating MetS-EVs, we constructed a ceRNA network. The ceRNAs that passed the specified criteria were all downregulated ceRNAs (lncRNA/mRNAs are downregulated, while miRNAs are upregulated). These networks presume that lncRNAs modulate directly expressional levels of miRNAs and mRNAs. The top 50 miRNAs putatively targeted by 191 lncRNAs were identified.

**FIGURE 6:**
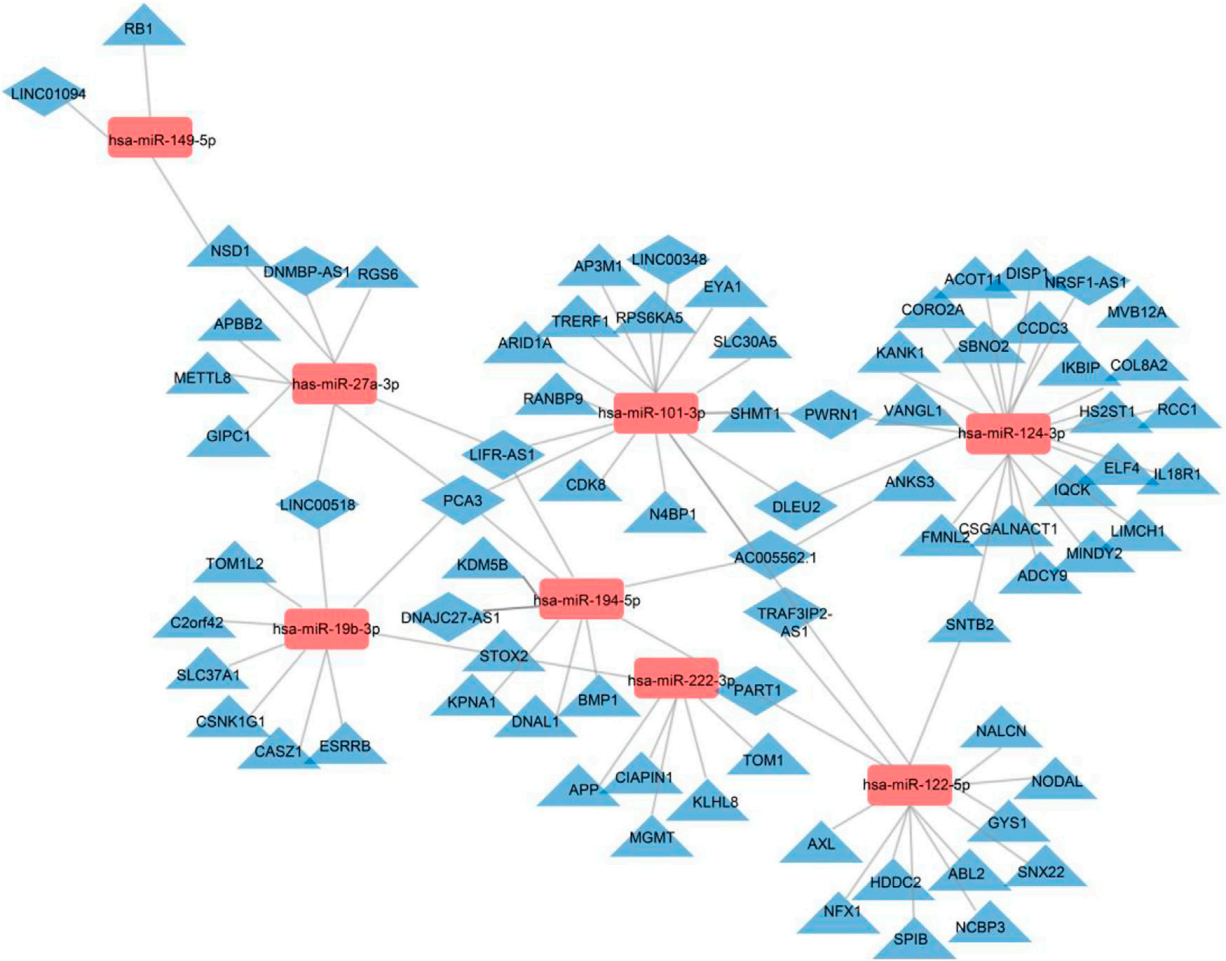
Regulatory network of the DE-mRNAs–DE-miRNAs–DE-lncRNAs in MetS. DE-mRNAs, DE-miRNAs, and DE-lncRNAs are indicated by triangles, rectangles, and diamonds, respectively. The red rectangles represent high expression levels, and blue triangles and diamonds represent low expression levels. Gray lines indicate lncRNA–miRNA–mRNA interactions.

**FIGURE 7:**
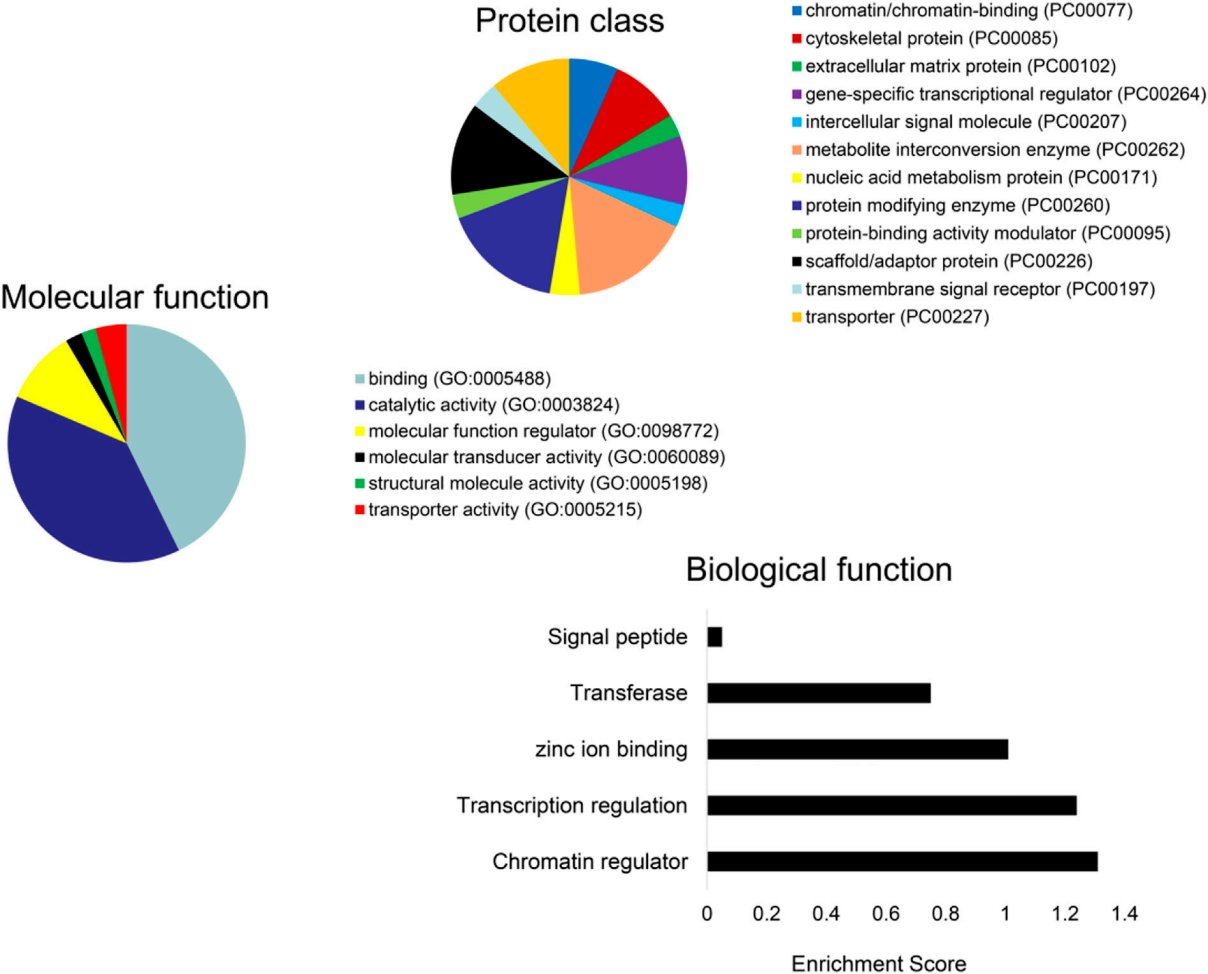
Functional analysis of the DE-mRNAs. PANTHER analysis of the predicted protein class **(A)** and molecular function **(B)** of DE-mRNAs. **(C)** Enrichment of functional pathways of the DE-mRNAs using DAVID 6.7.

Subsequently, we obtained the intersection of lncRNA–miRNA and miRNA–mRNA interactions by screening those RNAs and identified 26 DE-lncRNA–DE-miRNA interactions and 64 DE-miRNA–DE-mRNA interactions (Figure 6). Ultimately, 8 miRNAs, 13 lncRNAs, and 64 mRNAs were included in the ceRNA network. The identified lncRNA hub regulators included NR2F1-AS1, PART1, FOXC2-AS1, and PSMA3-AS1. Only few of the identified elements, such as miR-122 (Fredriksson et al., 2007), lncRNA FOXC2-AS1 (Lim et al., 2020), and glycogen synthase gene (Fredriksson et al., 2007), have been previously linked to MetS. Functional analysis identified most as cytoskeletal proteins and gene-specific transcriptional regulators that function in binding and catalytic activity. Additionally, many biological protein functions, including regulation of chromatin, transcription, zinc-ion binding, and transferase, are targeted by ceRNAs within those EVs (Figure 7).

## Discussion

This study employed high-throughput RNA sequencing to interrogate lncRNA, mRNA, and miRNA contents in circulating EVs in patients with MetS compared to Lean controls and constructed a MetS-circulating EV-specific ceRNA network. Our study demonstrated that metabolic syndrome alters the content of genes and miRNAs of circulating human EVs and revealed alterations in the ceRNA network which might be involved in the pathogenesis of MetS.

Constituting a leading cause of morbidity and mortality, MetS is a severe public health issue, which is strongly linked to cardiovascular events, as well as cancer. In the liver, non-alcoholic fatty liver disease is a well-recognized manifestation of MetS (Patel and Goyal, 2019), whereas in the kidney, MetS induces hyperfiltration and contributes to microvascular remodeling, podocyte injury, and mitochondrial dysfunction (Eirin et al., 2018). Congruently, in our study, patients with MetS showed elevated BUN, eGFR, ALT, and AST, which indicated early liver injury and renal hyperfiltration. Clearly, the onset and progress of MetS is a complex multi-factorial process. However, the molecular mechanisms activated in MetS remain obscure.

In addition to humoral factors, circulating EVs released from various tissues and organs reflect the status of their parental cells and may also mediate processes in target cells. We first characterized circulating EVs using accepted benchmarks. We then defined the expression of the top 30 DE-mRNAs, DE-miRNAs, and DE-lncRNAs in circulating MetS-EVs versus Lean-EVs and found that some of the top DE-RNAs have been implicated in conditions related to the pathogenesis or complications of MetS. The top DE-miRNA, miR-718, is involved in the regulation of vascular remodeling (Martínez-Micaelo et al., 2017), angiogenesis (Xue et al., 2014), and VEGF signaling (Leng et al., 2014), which are impacted by MetS. Of the top DE-mRNAs, GYS1 is involved in glucose metabolism (Xirouchaki et al., 2016), which is dysregulated in MetS. Validation studies using qPCR confirmed our RNA-Seq findings.

We found that many biological protein functions, including regulation of chromatin, transcription, zinc-ion binding, and transferase, are targeted by ceRNAs within those EVs. Regulation of chromatin modulates the access of binding proteins to DNA and histones and, in turn, gene expression, DNA replication, and DNA damage response (Li et al., 2018). Important zinc-binding proteins, such as zinc-finger genes, p53, or the Emi2 protein, play central roles in cancer and cell-cycle regulation. These observations suggest that MetS may impact the structure and function of modulators of central cellular processes.

In recent years, accumulating evidence has shown that bi-directional regulation between lncRNAs and miRNAs and their downstream target genes contributes to the development of many diseases, including MetS (Yuan et al., 2015). Studies on the underlying mechanisms by which lncRNAs regulate the onset and development of MetS have often focused on a single genomic event (Lan et al., 2016), which may not suffice to illustrate the complex involvement of lncRNAs or identify potential therapeutic targets for MetS.

LncRNAs are involved in tissue-specific transcriptional regulation (Cabili et al., 2011). While only a fraction of lncRNAs may be biologically relevant, at least some play an important role in regulating cellular function in metabolic diseases, such as cellular cholesterol metabolism. For example, lnc-HC negatively regulates cholesterol metabolism within hepatocytes through physical interaction with hnRNPA2B1 (Lan et al., 2016).

To examine the role of lncRNA in the ceRNA network of MetS, we constructed a MetS-specific network. In total, 1,389 DE-mRNAs, 191 DE-lncRNAs, and 138 DE-miRNAs were identified in circulating MetS-EVs compared to healthy controls. The miRcode and DIANA-LncBase databases were used to predict target lncRNAs of DE-miRNAs, and the miRTarBase database served to predict target mRNAs of DE-miRNAs. The MetS-specific ceRNA network was based on a total of 13 lncRNAs, 8 miRNAs, and 64 mRNAs. Some of these lncRNAs have been implicated in determination of a vascular smooth muscle cell phenotype, tumor progression, osteoarthritis, and Parkinson’s disease.

Interestingly, several identified lncRNA hub regulators are involved in reprogramming of tumor cells, whereas their involvement in MetS is yet to be reported. LncRNA NR2F1-AS1, which is linked with three miRNAs in the ceRNA network, promotes proliferation and migration yet suppresses apoptosis of thyroid cancer cells by sponging miRNA-338-3p (Guo et al., 2019). LncRNA PART1 regulates nucleus pulposus cell degeneration through the miR-93/metalloproteinase-2 pathway (Gao et al., 2020), lncRNA-DLEU2 was implicated in the growth and development of tumors, and lncRNA-PCA3 was linked especially to prostate cancer (Chunhua et al., 2018). LncRNA-PCA3 is linked with four miRNAs in the ceRNA network, including miR-27a-3p, miR-19b-3p, miR-101-3p, and miR-194-5p. LncRNA FOXC2-AS1 regulates cell proliferation and apoptosis through Akt/mTOR signaling and affects Notch signaling, a key regulator of the contractile phenotype of vascular smooth muscle cells (Lim et al., 2020). LncRNA PSMA3-AS1 promotes malignant phenotypes of esophageal cancer by modulating the miR-101/enhancer of zeste homolog-2 axis as a ceRNA (Qiu et al., 2020). Possibly, the cancer propensity of obesity might be partly traced to such DE-lncRNAs.

Analysis of the lncRNA–miRNA–mRNA crosstalk in the network uncovered miR-122, a key regulator of cholesterol and fatty-acid metabolism (Esau et al., 2006). In addition, miR-124 participates in inflammation, autophagy, mitochondrial function, and neurotransmission (Chu-Tan et al., 2018), whereas miR-149 mediates inhibition of cell proliferation, migration, and invasion and induces apoptosis (Yang et al., 2017). Therefore, DE-miRNAs have the potential to mediate some of the complications of MetS.

In addition, analysis of the pathway and enrichment of the DE-mRNAs in the ceRNA network revealed most of them to translate cytoskeletal proteins and gene-specific transcriptional regulators that function in binding and catalytic activity. They were involved in regulation of transcription, zinc-ion binding, and transferase. DE-mRNAs previously linked to metabolic diseases included glycogen synthase, Axl, and ginsenoside Rb1. Glycogen synthase has been associated with lower type 2 diabetes and features of MetS (Fenger et al., 2000) and Axl with the pathogenesis of obesity, insulin resistance, and inflammation (Li et al., 2019). In contrast, ginsenoside Rb1 improves insulin sensitivity (Yu et al., 2015) and lowers blood glucose by inhibiting p52 activation (Zhang et al., 2019).

Using next-generation sequencing analysis, we identified differential lncRNA, mRNA, and miRNA expression signatures in circulating EVs in MetS subjects compared with Lean human subjects and demonstrated the links among these DE-RNAs. Our study was limited by small sample sizes of relatively young patients, and larger studies in different ages and obesity levels are warranted. The MetS group was morbidly obese and exhibited significant complications of MetS, including hypertension, insulin resistance, dyslipidemia, and inflammation. Although defining the cargo of EVs derived from specific parent cells could provide valuable information, we focused on exploring the entire lncRNA cargo of all EVs that circulate in plasma. Few RNAs were significantly altered yet could drive meaningful signaling alterations in MetS individuals. Further studies are needed to determine the main determinants of these complications and the relationship between the ceRNA network and clinical features of MetS.

## Conclusion

This study suggests that MetS modifies the cargo of circulating EVs and the lncRNA-associated ceRNA network. Our findings support the notion that the ceRNA network might play essential roles in development and complications of MetS, including cancer. These findings shed light on potential biomarkers and mediators of MetS, which might serve as regulators and targets in the progression and treatment of MetS. Further studies are needed to elucidate the underlying mechanisms linking a dysregulated cargo of MetS-EVs and establish their roles in patients with MetS. Taken together, our observations demonstrate the role of MetS in altering the lncRNA cargo of circulating EVs. Importantly, these findings may contribute to identifying novel biomarkers in MetS.

## Data Availability

The datasets presented in this study can be found in online repositories. The names of the repository/repositories and accession number(s) can be found below: https://www.ncbi.nlm.nih.gov/bioproject/?term=PRJNA672664, PRJNA672664 and https://www.ncbi.nlm.nih.gov/geo/, GSE166474.

## Abbreviations

MetS, metabolic syndrome; EVs, extracellular vesicles; miRNA, micro-RNA; eGFR, estimated glomerular filtration rate; ALT, alanine aminotransferase; AST, aspartate aminotransferase; ALP, alkaline phosphatase; PRA, plasma renin activity.
